# The Nagoya protocol and research on emerging infectious diseases

**DOI:** 10.2471/BLT.19.232173

**Published:** 2019-06-01

**Authors:** Sascha Knauf, Lena Abel, Luisa K Hallmaier-Wacker

**Affiliations:** a Neglected Tropical Diseases Work Group, Infection Biology Unit, German Primate Center, Leibniz Institute for Primate Research, Kellnerweg 4, 37077 Göttingen, Germany.

The *Nagoya protocol on access to genetic resources and the fair and equitable sharing of benefits arising from their utilization to the Convention on Biological Diversity*^1^ came into effect in 2014. The protocol aims to regulate research on genetic resources: tissue samples, DNA sequences, cultures or specimens, and provide a framework for benefit-sharing with the countries of origin when commercially-viable products result. These genetic resources are subject to national legislation on the conditions of collection, storage and use.

The way that the Nagoya protocol is implemented can hinder international research collaborations.^2^^,^^3^ In our experience, microbiologists need to routinely share microorganisms, deposit isolates in culture collections and submit their research data to publicly-accessible databases. The Nagoya protocol seems to have created new barriers to the deposit of samples and the availability of sequences.^4^

Most microorganisms are wide-spread, of unlimited supply and of no monetary value.^3^ Less than 0.1% of the estimated species of bacteria^5^ and less than 2.0% of estimated fungal species^3^ have been studied to date. Of these, a relatively small number of pathogenic microorganisms are of outsized importance due to their potential for causing crop failure, human and animal disease and deaths.^6^ Article 8b of the Nagoya protocol recommends that parties “Pay due regard to cases of present or imminent emergencies that threaten or damage human, animal or plant health, as determined nationally or internationally.”^1^ The protocol does not specify how its parties shall implement these considerations.^7^ Five years into protocol implementation, 56% (42/75) of its parties report paying attention to cases of present or imminent emergencies that threaten or damage human, animal or plant health.^8^ However, only 36% (36/75) of the parties consider it is necessary to allow exceptions or fast-track options for research on pathogens and/or emerging infectious diseases, and of these countries, very few have exceptions published.^8^ Obtaining prior informed consent and mutually agreed terms between providers and users of any given biological sample takes from several weeks to months. These delays conflict with current research funding as many multicentre studies are unable to compensate for the delays and increased workload that is associated with such regulations.

We agree that the sovereignty of any country and its right to regulate the use of its biological resources must be upheld. Global epidemic preparedness requires the continuous detection of pathogens and the monitoring of known and potential human and animal reservoirs systems^9^ to detect emerging diseases early. Pathogens spread regardless of national borders and control measures require partnerships between countries and research institutions. Constrained partnerships in non-commercial research can harm the global interest of protecting against emerging infectious diseases. Long negotiations to obtain specimens for research potentially increases the risk of infectious diseases spreading in populations that have no immunity. 

The One Health approach, where human, animal and environmental health are considered as inevitably linked, is widely accepted as a pillar of public health.^10^ The Nagoya protocol, in its current form, does not sufficiently recognize the need for global collaborative research on genetic resources that have the potential to become a risk to public health. We believe that the protocol neglects the non-commercial basic research on microorganisms needed for the global capacity to prevent epidemics. Therefore, we request that parties to the Convention consider simplified measures for non-commercial research, in their legislation on access and benefit sharing, including exceptions or fast track options for research on pathogens.

## References

[1] Nagoya protocol on access to genetic resources and the fair and equitable sharing of benefits arising from their utilization to the convention on biological diversity. Montreal: Secretariat of the Convention on Biological Diversity; 2011. Available from https://www.cbd.int/abs/doc/protocol/nagoya-protocol-en.pdf [cited 2018 Dec 22].

[2] Deplazes-Zemp A, Abiven S, Schaber P, Schaepman M, Schaepman-Strub G, Schmid B, et al. The Nagoya Protocol could backfire on the Global South. Nat Ecol Evol. 2018 6;2(6):917–9. 10.1038/s41559-018-0561-z 29760439

[3] Overmann J, Scholz AH. Microbiological research under the Nagoya Protocol: facts and fiction. Trends Microbiol. 2017 2;25(2):85–8. 10.1016/j.tim.2016.11.001 27887771

[4] Dedeurwaerdere T, Melindi-Ghidi P, Broggiato A. Global scientific research commons under the Nagoya Protocol: towards a collaborative economy model for the sharing of basic research assets. Environ Sci Policy. 2016 1;55:1–10. 10.1016/j.envsci.2015.08.006 28149197 PMC5268345

[5] Overmann J. Principles of enrichment, isolation, cultivation, and preservation of prokaryotes. In: Rosenberg E, DeLong EF, Lory S, Stackebrandt E, Thompson F, editors. The prokaryotes. Berlin, Heidelberg: Springer; 2013 10.1007/978-3-642-30194-0_7

[6] Morse SS, Mazet JA, Woolhouse M, Parrish CR, Carroll D, Karesh WB, et al. Prediction and prevention of the next pandemic zoonosis. Lancet. 2012 12 1;380(9857):1956–65. 10.1016/S0140-6736(12)61684-5 23200504 PMC3712877

[7] Buck M, Hamilton C. The Nagoya Protocol on access to genetic resources and the fair and equitable sharing of benefits arising from their utilization to the Convention on Biological Diversity. Rev Eur Comp Int Environ Law. 2011;20(1):47–61. 10.1111/j.1467-9388.2011.00703.x

[8] Analysis of information contained in the interim national reports and information published in the access and benefit-sharing clearing house. Montreal: Convention on Biological Diversity, United Nations; 2018. Available from https://www.cbd.int/doc/c/767b/a3b0/e4934613a1a3fd1116b1c89a/sbi-02-inf-03-en.pdf [cited 2018 Dec 22].

[9] Hallmaier-Wacker LK, Munster VJ, Knauf S. Disease reservoirs: from conceptual frameworks to applicable criteria. Emerg Microbes Infect. 2017 09 6;6(9):e79. 28874791 10.1038/emi.2017.65 PMC5625316

[10] Kahn LH, Kaplan B, Monath TP, Steele JH. Teaching “one medicine, one health”. Am J Med. 2008 3;121(3):169–70. 10.1016/j.amjmed.2007.09.023 18328295 PMC7119384

